# London

**Published:** 1855-06

**Authors:** 


### LONDON.

THE following is an abstract of the Epidemics occurring in London during the months of March, April, and May, 1855, as indicated by the Registrar-General's Report:—

*Scarlet Fever* presented a weekly mortality exceeding the average of the preceding ten corresponding periods. The deaths varied from 35 in the weeks ending March 17 and 24 and May 12, to 52 in the week ending May 26. The west, north, central, and south districts suffered pretty uniformly in proportion to the population: the deaths in the west district amounting to 88, or 1 in 4277 inhabitants; in the north to 123, or 1 in 3987; in the central to 98, or 1 in 4013; in the south to 145, or 1 in 4253. In the east districts the mortality was 96, or 1 in 5051.

*Measles.*—The mortality from this disease from the third week of the period, was uniformly below the average of ten years, latterly scarcely exceeding one half. Out of 201 deaths, 95 occurred in the east districts, being in the proportion of 1 to 5111 inhabitants. In the west districts the deaths from measles were only 8, or 1 in 47,053. The greatest number of deaths was 22, in the week ending March 10; the lowest 9, in the week ending May 12.

*Small-pox.*—The deaths from this disease amounted to 300. Of these, 95 occurred in the north districts, being 1 in 5162 inhabitants; 72 in the east, or 1 in 6743; 71 in the south, or 1 in 8685; 36 in the central, or 1 in 10,368; and 26 in the west districts, or 1 in 14,478. The weekly mortality from small-pox varied much, presenting the following numbers in the thirteen weeks of the period: 23, 23, 16, 11, 21, 16, 19, 30, 15, 28, 45, 29, and 24. The average mortality from this disease varied in the corresponding period of the preceding ten years, from 13 to 17; hence it will be seen that the number of deaths was generally much above, and in one week triple of, the average. In the week ending March 17, 7 deaths, and in the week ending May 19, 11 deaths occurred in the Small-pox Hospital: all of the former, and 9 of the latter, occurred in persons who had not been vaccinated.

*Hooping Cough* was fatal in 782 cases. Of these, 151 occurred in the north districts, or 1 in 2493; 189 in the west, or 1 in 2595; 192 in the south, or 1 in 3211; 121 in the central, or 1 in 3250; and 129 in the east districts, or 1 in 3764. It will be seen that this disease was pretty equally diffused throughout London. The greatest amounts of mortality from it was in the first five weeks of the period, when it reached from 74 to 78; after this it sank to a weekly average of about 50 (slightly above the decennial average) till it reached 41 in the week ending June 30.

*Croup* was much less prevalent and fatal than during the preceding three months: although the deaths from it continued to the end of the period above the average. The greatest number of deaths occurred in the week ending April 14, when 18 occurred; in the preceding and succeeding weeks, the numbers were respectively 11 and 7. The ordinary weekly mortality from croup varied irregularly from 5 to 12.

*Influenza.*—The total number of deaths from this disease during the three months embraced in this report was 37, of which 23 occurred in the first three weeks. Subsequently to that period, the mortality from influenza has in no week exceeded 2, and has been below—sometimes only one-third of—the average decennial number of deaths. With 14 exceptions, the deaths from influenza occurred in persons above 60 years of age.

*Erysipelas.*—The number of deaths from this malady varied from 4 to 11; on the whole, the mortality was below the average. The greatest number of deaths occurred in the first five weeks.

*Ague.*—Six persons died from this disease during the period.

*Remittent Fever.*—The weekly mortality from this disease varied irregularly from 1 to 5, being generally from 1 to 3.

*Diarrhœa* proved fatal in 22 and 21 cases in the weeks respectively ending March 10 and April 28. The weekly mortality varied generally from 11 to 15. The total mortality was 192, viz., 21 in the west districts, 30 in the north, 23 in the central, 43 in the east, and 75 in the south districts.

*Dysentery* was fatal in 5 cases in each of the weeks ending March 24, May 12 and 26. In all, 30 deaths from this disease occurred.

*Typhus* presented a weekly mortality varying from 30 to 49. The total number of deaths was 518. It was most fatal in the east and south districts: the deaths being, in the east districts 140, or 1 in 3468 inhabitants; and in the south districts 165, or 1 in 3737. The mortality in the central districts amounted to 61, in the west to 74, and in the north districts to 78.

*Puerperal Fever* presented a rate of mortality somewhat above the average. During the quarter 56 persons died of this disease—8 in the week ending March 17.

*Carbuncle* caused death in 10 cases. In six weeks there were no deaths; but in one (that ending May 26) three cases occurred.

